# Supplementary material for: Venomous Noodles: The Evolution of Toxins in Nemertea through Positive Selection and Gene Duplication
Source: Toxins (Basel). 2023 Nov 12;15(11):650. doi: 10.3390/toxins15110650 (PMC10674772; doi:10.3390/toxins15110650)
Supplement: Supplementary file 1 [file toxins-15-00650-s001.zip › toxins-2612471-supplementary figures and tables S1, S5, S6.pdf]

Article

Venomous Noodles: Evolution of Toxins in Nemertea through Positive Selection and Gene Duplication

Gabriel Gonzalez Sonoda [1], Eric de Castro Tobaruela [2], Jon Norenburg [3], João Paulo Fabi [4] and Sónia C. S. Andrade [5,*]

Supplementar file

Supplementar Figures:

- Figure S1: BUSCO assessment results
- Figure S2: Occupancy Matrix
- Figure S3: Venn Diagram
- Figure S4: Heatmap of summed protein area for *Lineus sanguineus* (a) and *Nemertopsis berthalutzae* (b)
- Figure S5: Antistasin coverage
- Figure S6: CODEML analyses for *Cytotoxin-A.*
- Figure S7: CODEML analyses for *Scoloptoxin-SD976.*
- Figure S8: CODEML analyses for *Alpha-Nemertide.*
- Figure S9: CODEML analyses for *Beta-Nemertide.*
- Figure S10: CODEML analyses for *U-Nemertotoxin-3.*
- Figure S11: CODEML analyses for *Alpha-Ktx-like.*
- Figure S12: Alignments of *Cytotoxin-A* transcripts to *Lineus longissimus* genome.
- Figure S13:Alignments of *Scoloptoxin-SD976* transcripts to *Lineus longissimus* genome.
- Figure S14: Alignments of the remaining toxins in to *Lineus longissimus* genome.
- Figure S15: Orthofinder duplication analyses.
- Figure S16: Toxin orthogroups contribution to duplications in each node.
- Figure S17: Analyses pipeline

Supplementar tables

- Table S1: Statistics of the assembled transcriptomes. RIN: RNA integrity number.
- Table S2: *Lineus sanguineus* toxins

- Table S3: *Nemertopsis pamelaroeae* toxins
- Table S4: *Ototyphlonemertes erneba* toxins
- Table S5: Number of gene copies resulting from duplication events inferred from Tree reconciliation method and number of Loci inferred from alignments to *Lineus longissimus* genome for each orthogroup
- Table S6: OTUs used for phylogeny construction and selection test and their respective accession code. Outgroup samples are marked with asterisks. Samples from the present work are in bold.

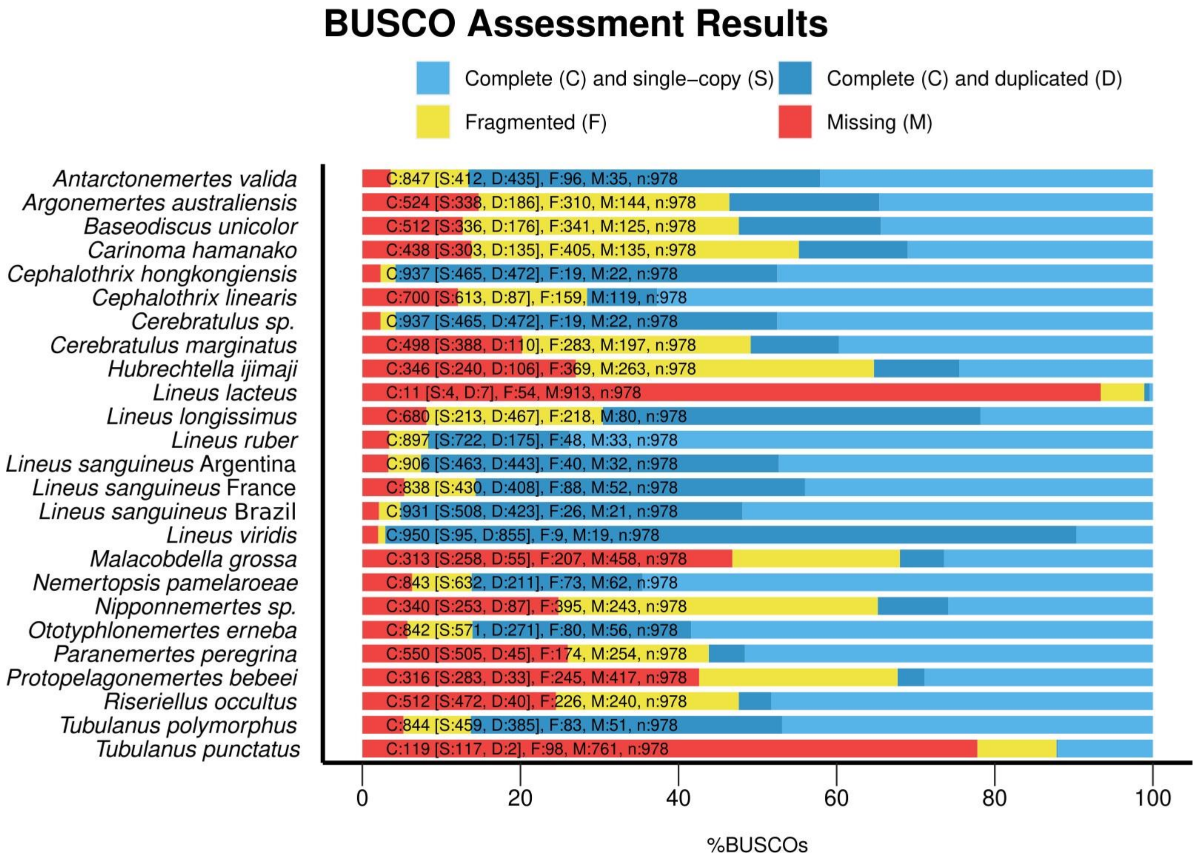

Figure S1. BUSCO assessment results. The used database was the odb9 for metazoans conserved genes (n=978 genes), hence each hit corresponds to a conserved metazoan gene.

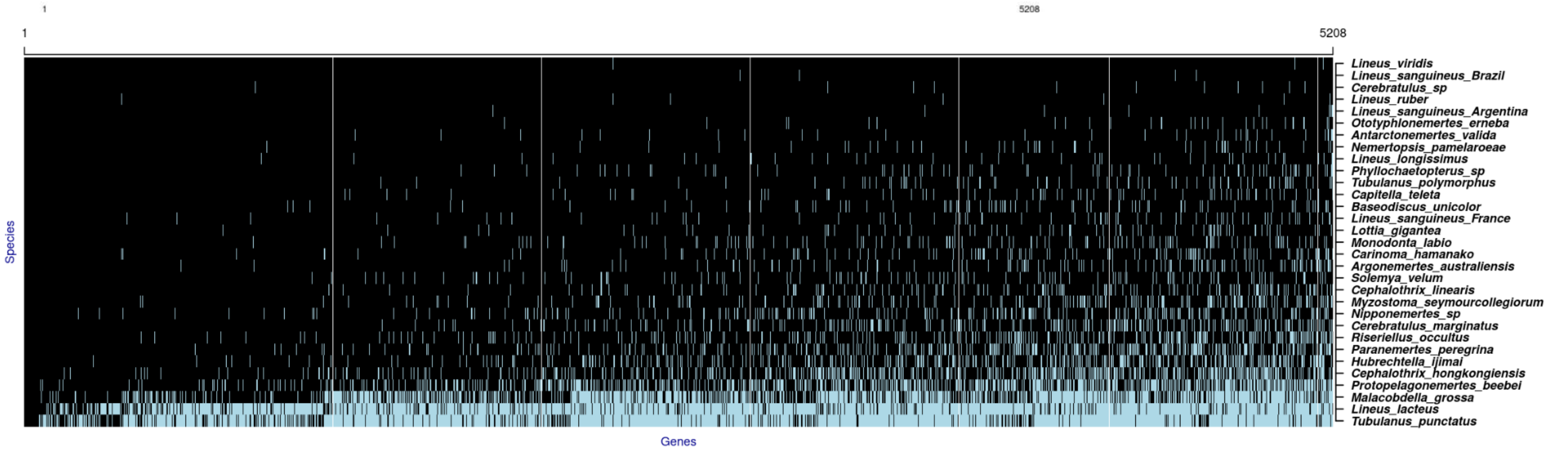

Figure S2: Occupancy matrix for the 5,208 genes used in the phylogenomic step. Dark tiles represent orthogroups present. Species are ordered from highest to the lowest occupancy.

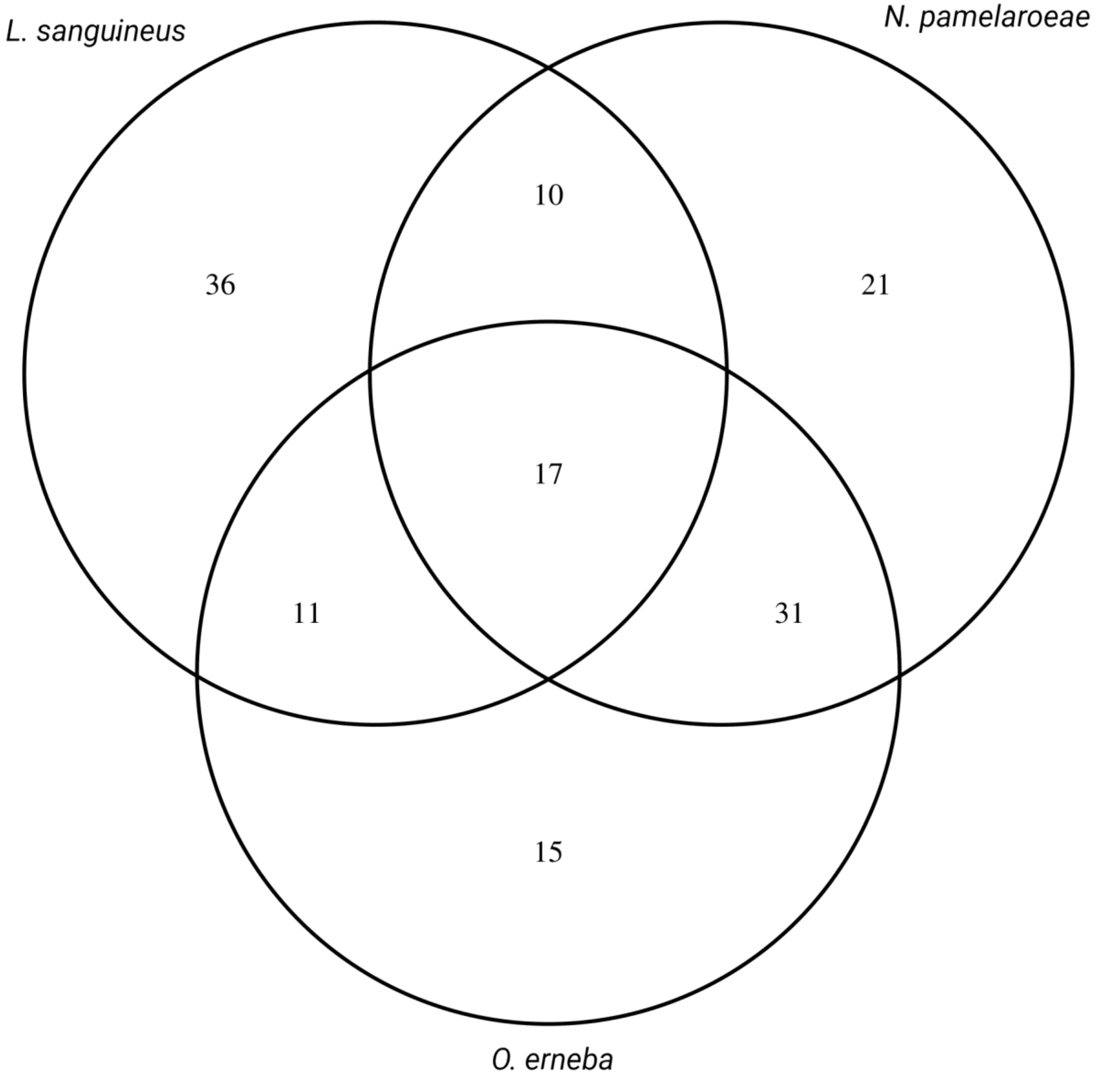

Figure S3: Venn diagram of putative toxin best hits found in the transcriptome of the three species sequenced in this study.

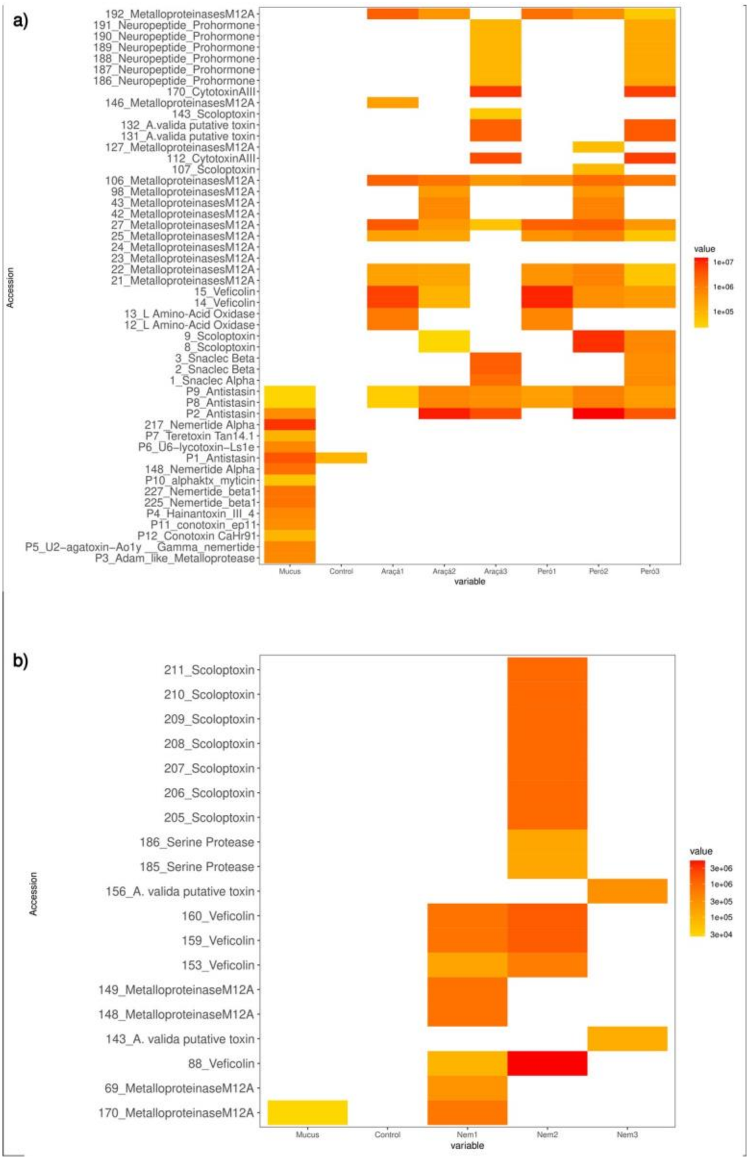

Figure S4: Abundance of toxins in the proteomic sample, indirectly measured by the summed area of peptide peaks in the HPLC chromatogram, calculated by PEAKS Studio Xpro, for mucus and body fractions replicates from a) *Lineus sanguineus* and b) *Nemertopsis berthalutzae*. Ar: Body sample of *L. sanguineus* collected in Araçá; P: Body sample of *L. sanguineus* collected in Peró; Nem: Body sample of *N. berthalutzae* collected in Praia Grande; 1: Fraction of proteins with 50 KDa or more; 2: Fraction of proteins between 20KDa and 50KDa; 3: Fraction of proteins of 20KDa or lower; IDs between parentheses correspond to IDs from tables S3 and S4.

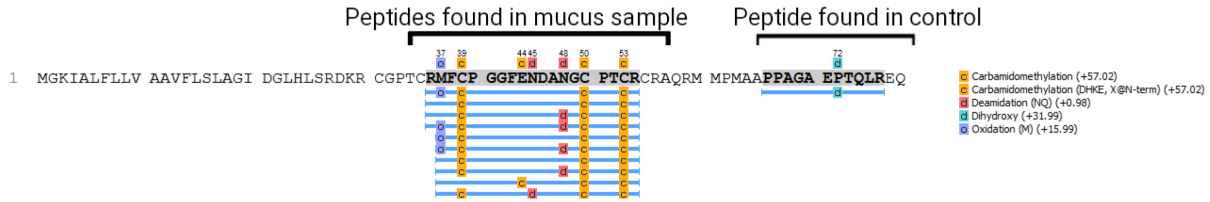

Figure S5: Antistasin (L92) protein sequence coverage by HPLC-MS/MS of *Antistasin-like protein*. Peptides found are represented by light blue lines. Detected amino-acid modifications are indicated by boxes.

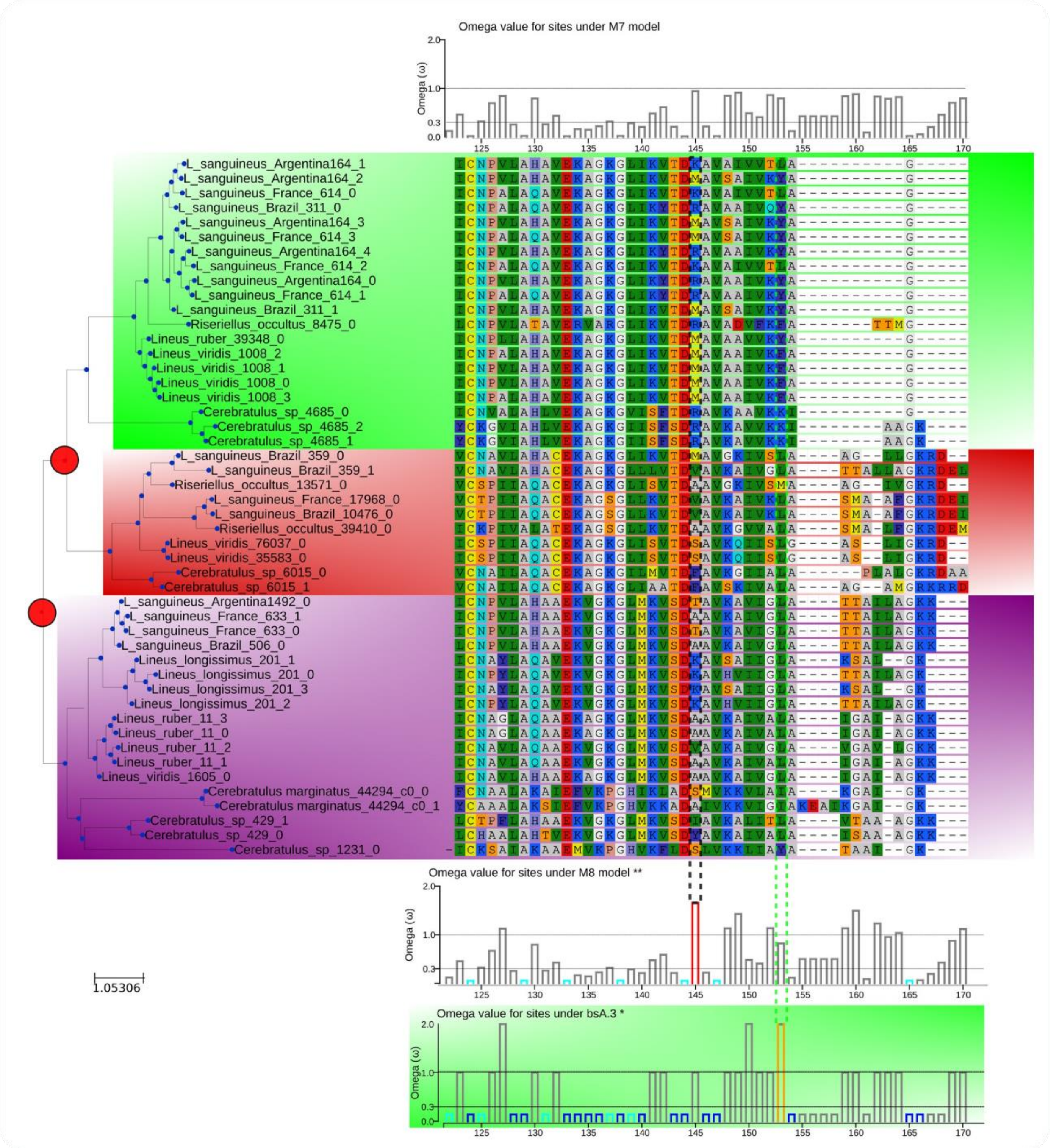

Figure S6: Gene tree reconciliation, alignment and omega value for sites of the Cytotoxin-A Orthogroup. The reconciled gene tree (left) was inferred by comparing the maximum-likelihood gene tree and the species tree using DLCpar to identify the most parsimonious history of gene duplications (red circles in the tree), losses and deep coalescence events. Colored branches were tested for positive selection with the branch-site model. Orange bars: $p<0.05$ for $dN/dS>1$, red bars: $p<0.01$ for dN/dS, light blue: $p<0.05$ for $dN/dS<1$ and dark blue: $p<0.01$ for $dN/dS<1$.

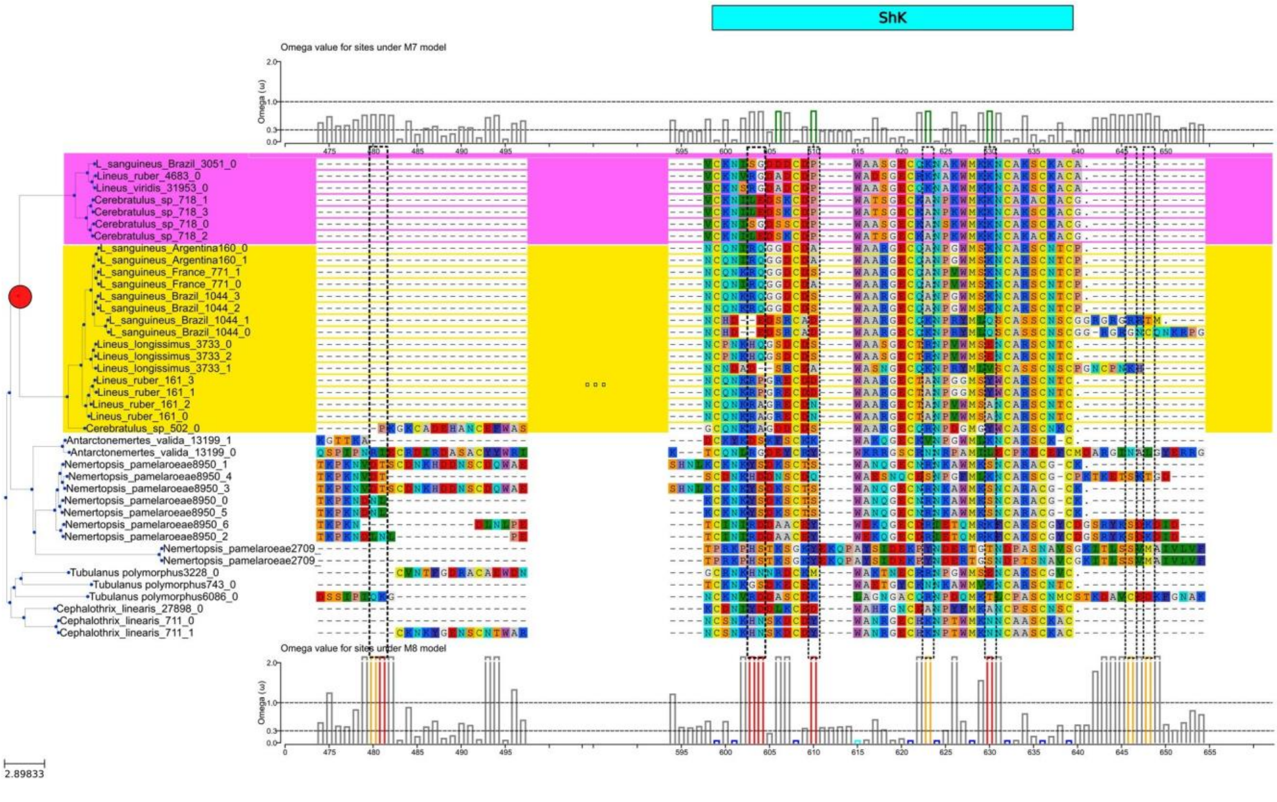

Figure S7: Gene tree reconciliation, alignment and omega value for sites of the *Scoloptoxin SDD976-like* curated gene family. Legend goes as in FigureS 12. ShK: ShK domain-like.

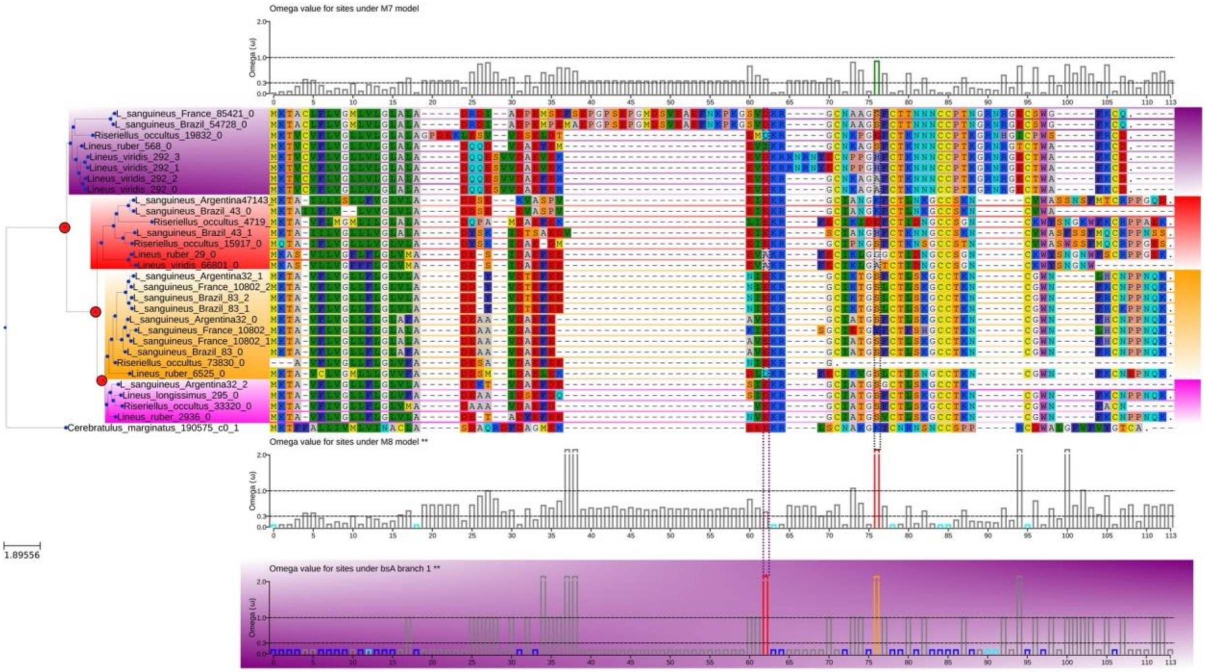

Figure S8: Gene tree reconciliation, alignment and omega value for sites of the *Alpha-Nemertide* gene family. Legend goes as in Figure 2.

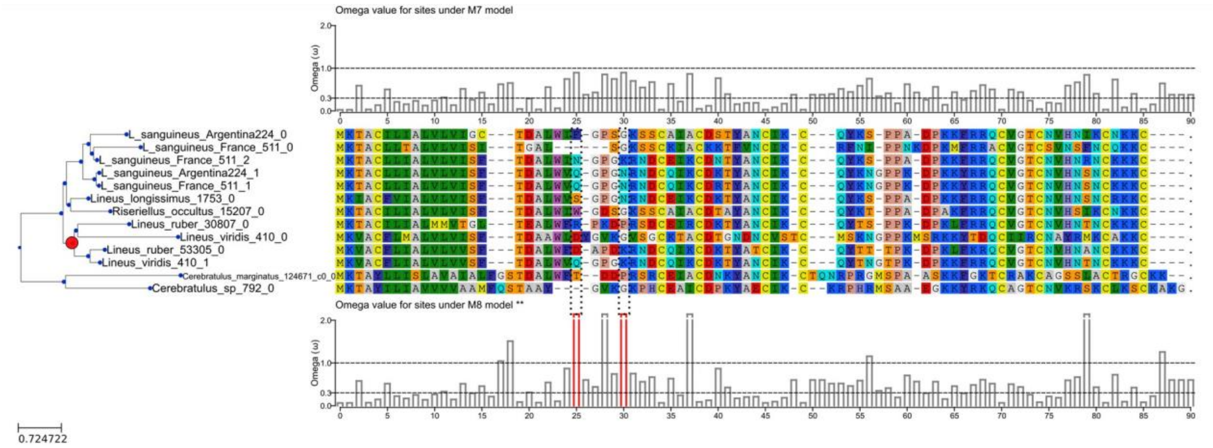

Figure S9: Gene tree reconciliation, alignment and omega value for sites of the *Beta-Nemertide* gene family. Legend goes as in Figure S12.

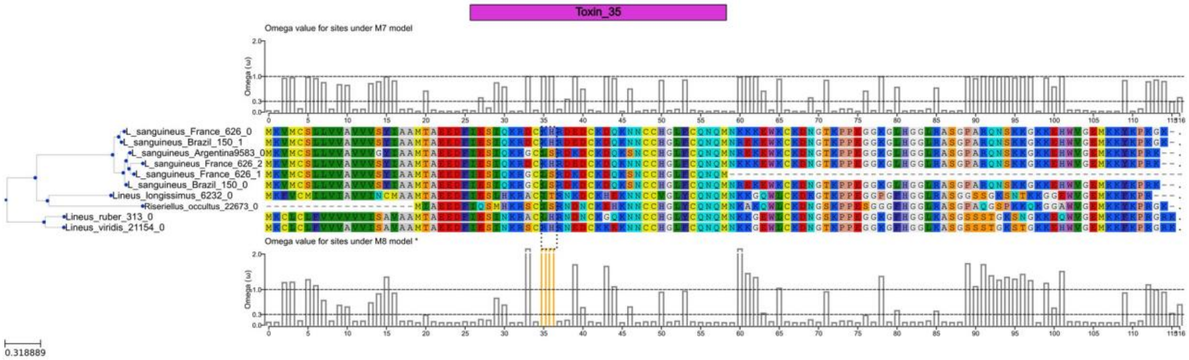

Figure S10: Gene tree reconciliation, alignment and omega value for sites of the *U-Nemertotoxin-3* gene family. Legend goes as in Figure 2. Toxin_35: Toxin with inhibitor cystine knot ICK or Knottin scaffold.

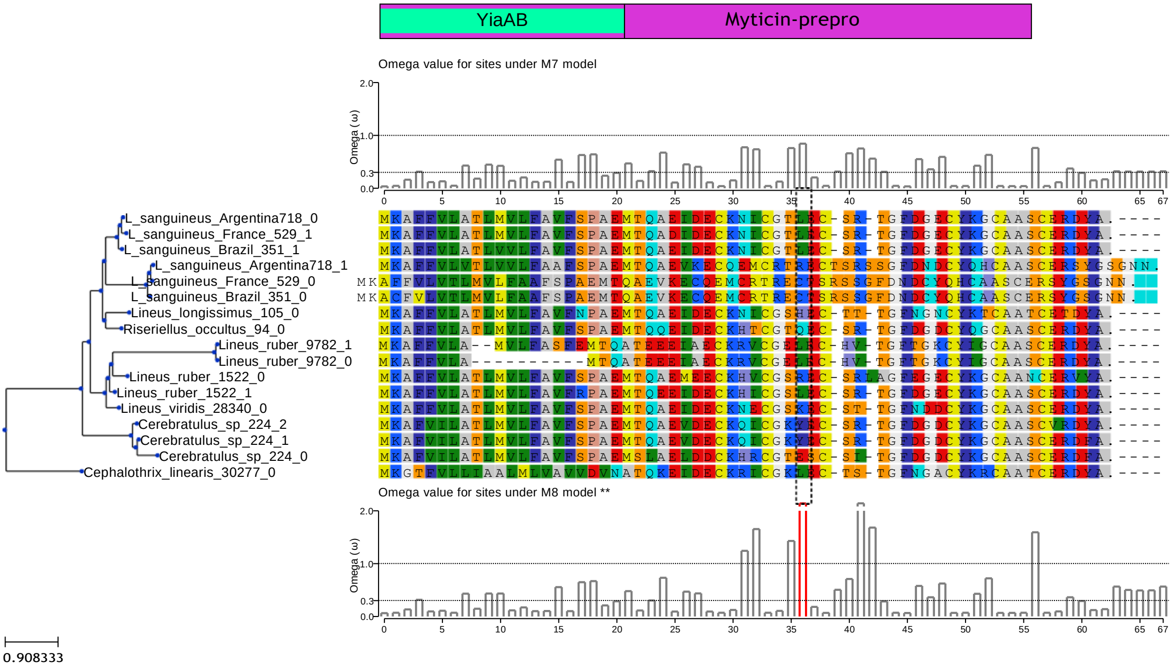

Figure S11: Gene tree reconciliation, alignment and omega value for sites of the *alpha-KTx/Beta-defensin/myticin* gene family. Legend goes as in Figure S12. YiaAB: yiaA/B two helix domain; Myticin-prepro:Myticin pre-proprotein from the mussel; Toxin_2: Scorpion short toxin only identified in the clade containing L_sanguineus_Brazil_351_0, L_sanguineus_France_529_0, L_sanguineus_Argentina718_1.

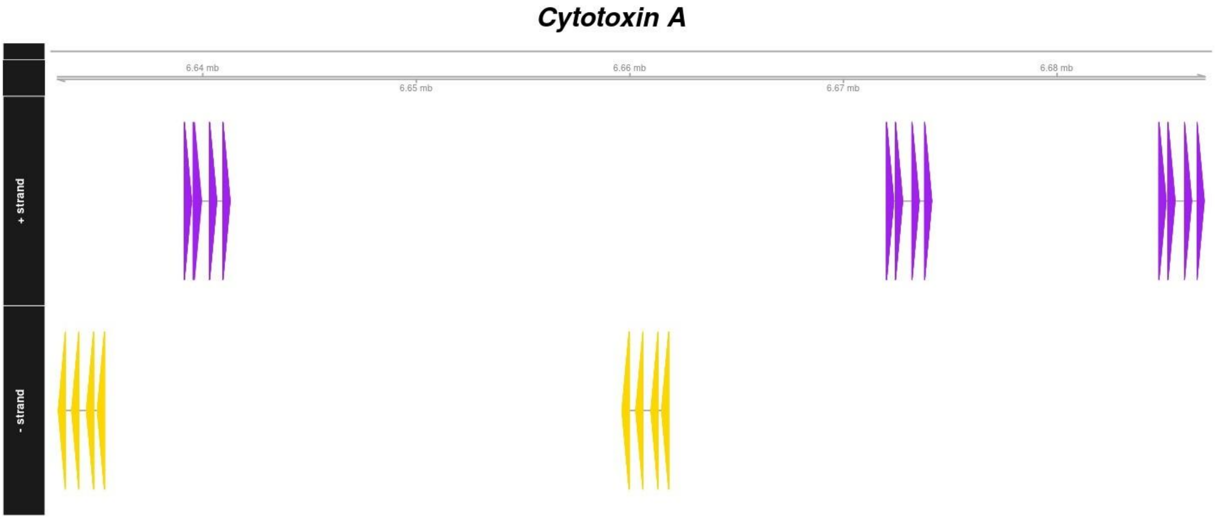

Figure S12: Genomic regions of *Lineus longissimus* aligned to Cytotoxin-A transcripts. Different colors represent the different strands. All the reported alignments covered 100% of the query transcript.

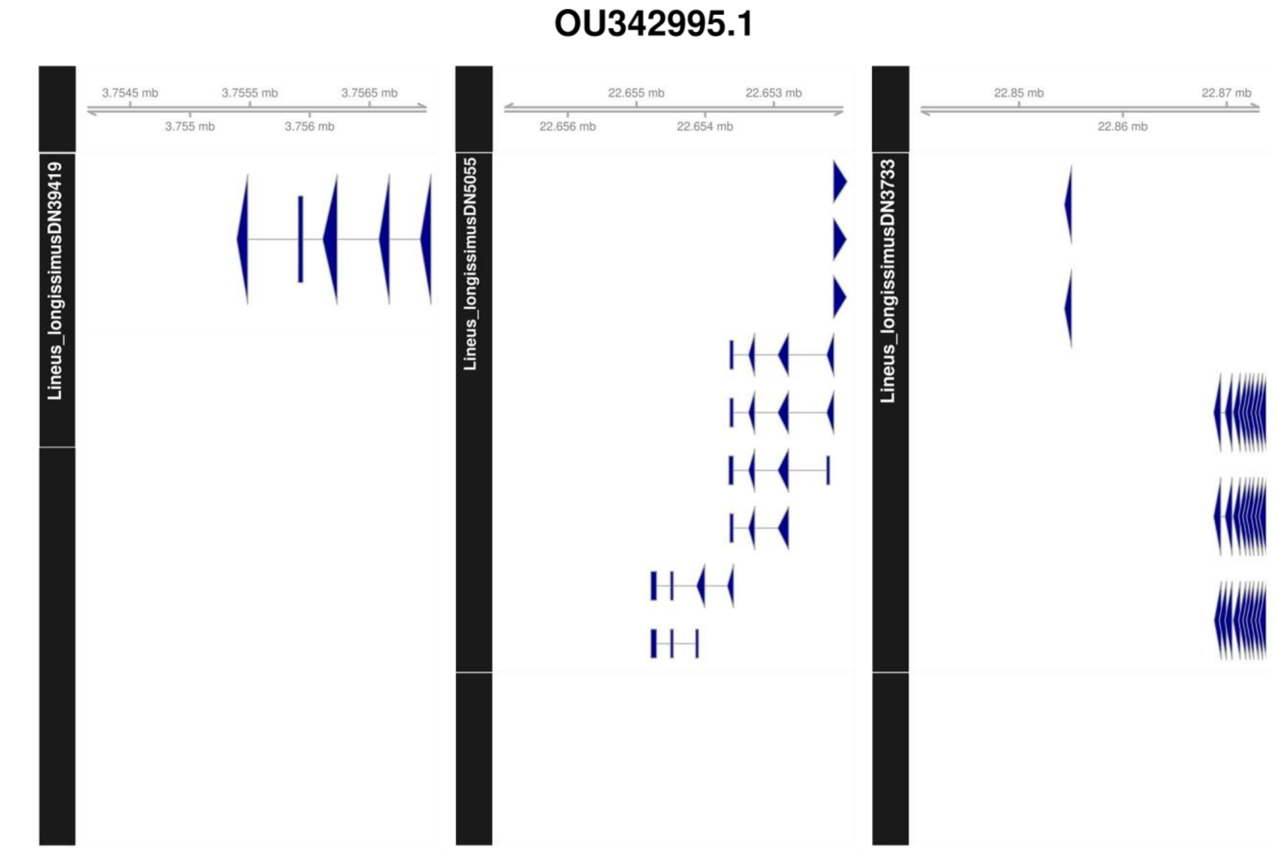

Figure S13: Genomic region containing regions identified as homologous to the Scoloptoxin-SD976-like putative toxins. The only *Lineus longissimus* transcript included in the final Scoloptoxin-SD976-like alignment was the LlongTRINITY_DN3733. OU342995.1: Scaffold name from *Lineus longissimus* genome assembly.

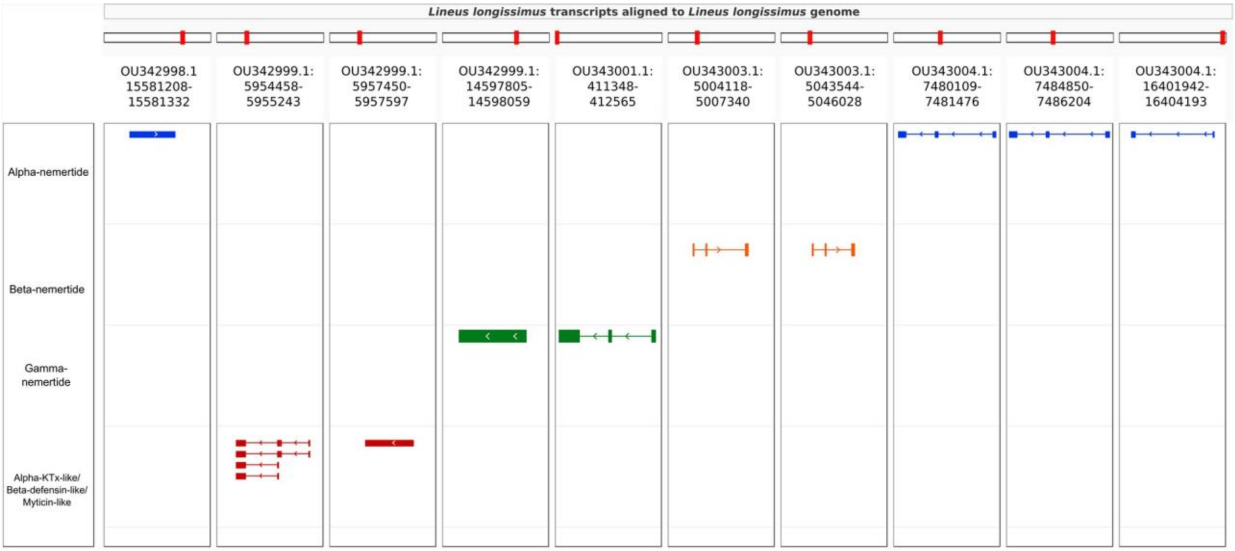

Figure S14: Genomic regions of *Lineus longissimus* containing aligned to the transcripts of the analyzed toxins present in the mucus of *Lineus sanguineus*. The header of each column contains the scaffold name and the range of represented bases. Different colors represent the different toxins prospected.

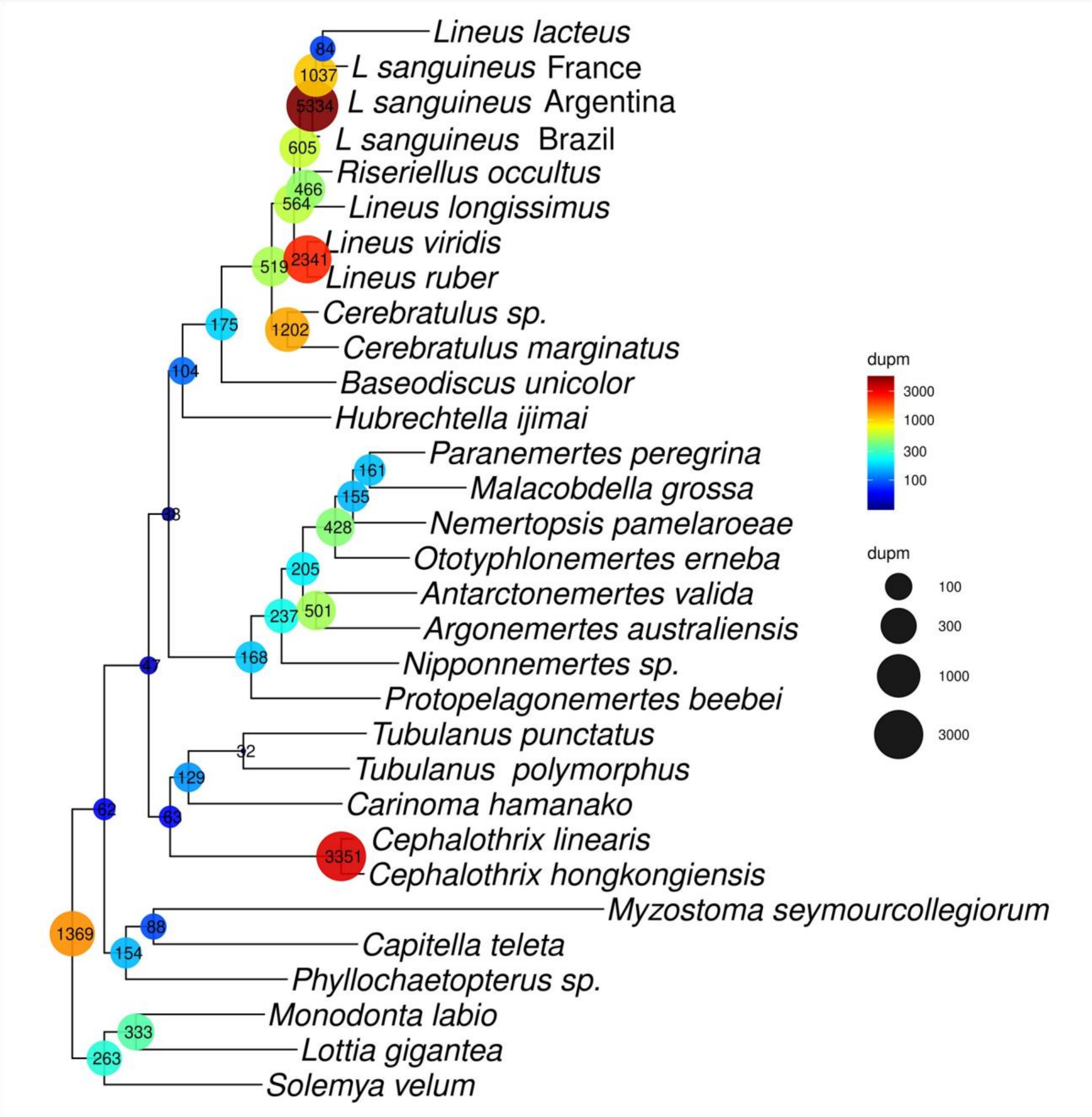

Figure S15: Gene duplications per node identified with OrthoFinder and duplication support value higher than 50%. A support of at least 50% means that at least half of the species present in given node are also present at the two direct children nodes.

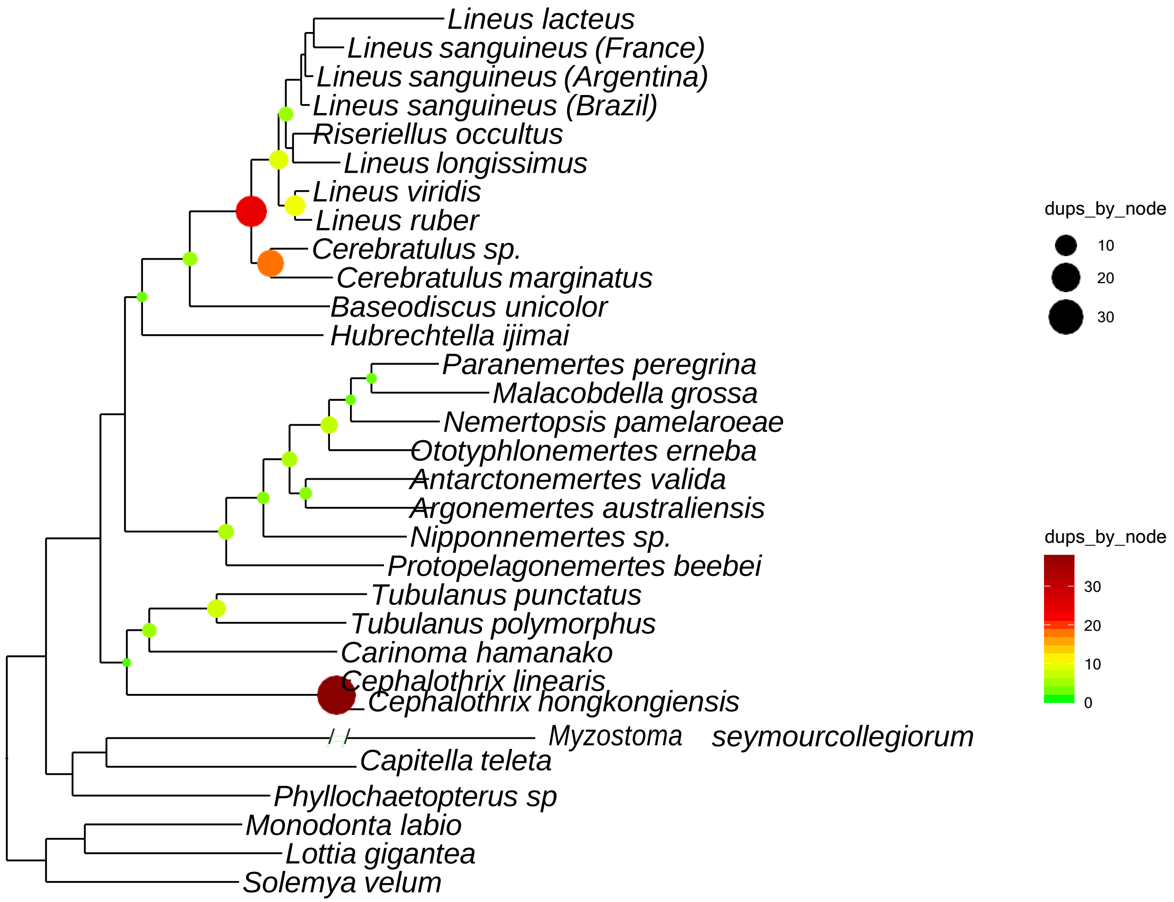

Figure S16: Duplications in orthogroups containing putative toxins for each node in the species tree, with support value higher than 50%. Duplication events were identified with OrthoFinder.

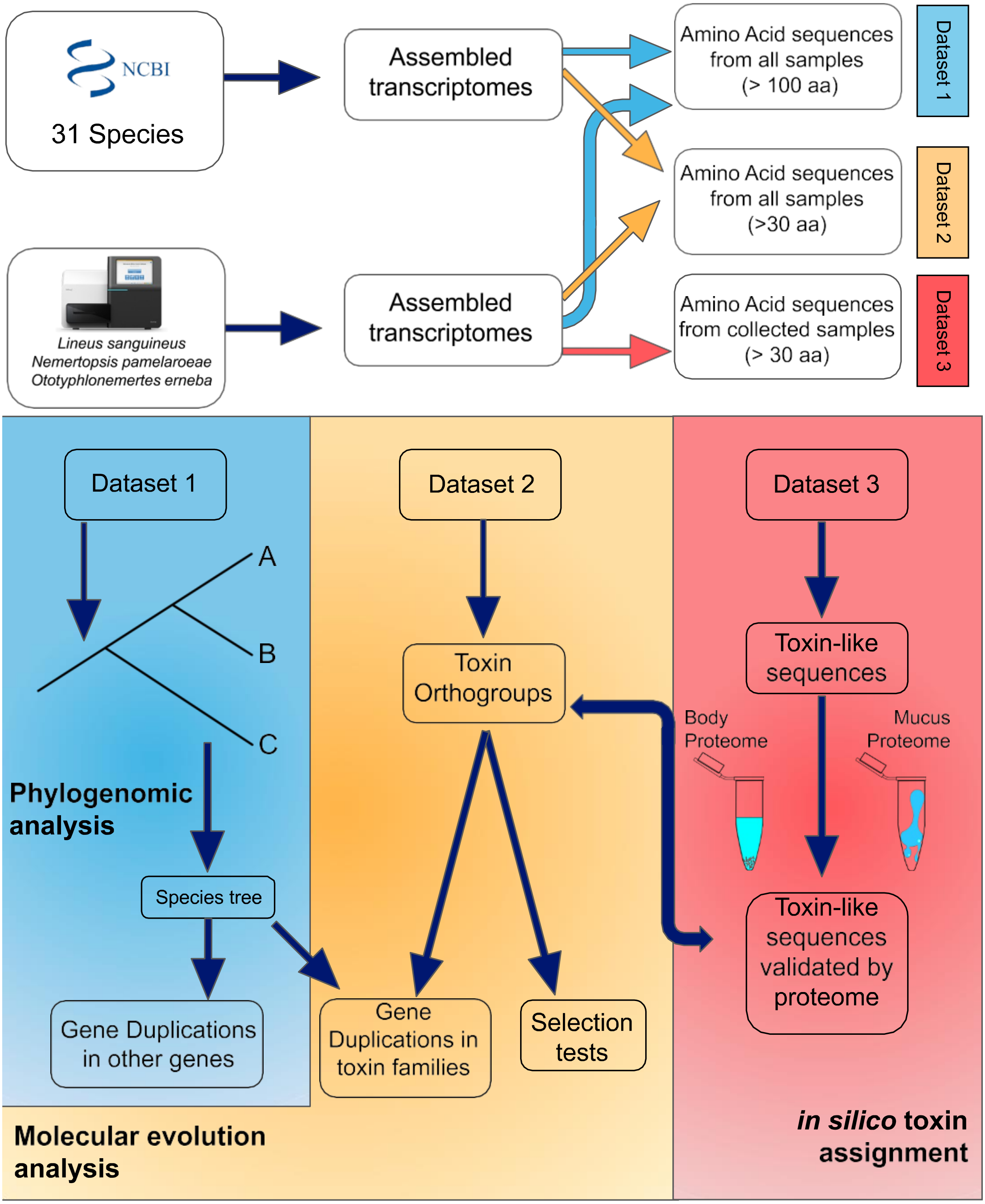

Figure S17: Analyses pipeline. Dataset 1 was used to infer a well supported species tree and estimate overall gene duplication rates. Dataset 2 was used to infer toxin orthogroups, from which gene duplications analyses and selection tests were performed. Dataset 3 was used to search for proteins in the mucus and body proteomes from the analyzed species, in order to identify and validate putative toxins. aa= amino acid.

Table S1: Statistics of the assembled transcriptomes. RIN: RNA integrity number.

Species	RIN	Number of raw reads	Number of paired filtered reads	#contigs	#contigs >500pb	#contigs >1000pb	longest contig	N50
Lineus sanguineus	7.70	13.441.397	12.229.480	113.957	65.600	44.455	30.915	3.167
Ototyphlonemertes erneba	7.30	12.859.411	11.688.148	99.617	46.875	28.177	34.319	2.417
Nemertopsis pamelaroeae	8.60	12.106.267	11.008.779	54.385	26.684	15.019	19.971	2.079

Tables S2-S4 are available in TableS2_S4.xls

Table S5: Number of gene copies resulting from duplication events inferred from Tree reconciliation method and number of Loci inferred from alignments to *Lineus longissimus* genome for each orthogroup.

Orthogroup	Tree reconciliation Method	Genome Alignment
Cytotoxin A	3	5
Scoloptoxin SD976-like	2	2 *
Alpha Nemertide	4	4
Beta Nemertide	2	2
Gamma Nemertide	1	2
KTX-like	1	2

*Only the transcripts kept by MaxAlign were aligned to the genome

Table S6: OTUs used for phylogeny construction and selection test and their respective accession code. Outgroup samples are marked with asterisks.
Samples from the present work are in bold.

OTU	SRR, SRX or GCA (When specified)
Antarctonemertes valida	PRJNA485632
Argonemertes australiensis	PRJNA254358
Baseodiscus unicolor	PRJNA322119
Carinoma hamanako	PRJNA254071
Cephalothrix hongkongiensis	PRJNA181263
Cephalothrix linearis	PRJNA245790
Cerebratulus marginatus	PRJNA181261
Cerebratulus sp.	PRJNA275078
Hubrechtella ijimai	PRJNA254167
Lineus lacteus	PRJNA530965
Lineus longissimus	PRJNA330781
Lineus ruber	PRJNA249058
Lineus sanguineus (Argentina)	PRJNA322119
Lineus sanguineus (Brazil, BR)	**PRJNA952238**
Lineus sanguineus (France)	PRJNA322119
Lineus viridis	PRJNA322119
Malacobdella grossa	PRJNA254359
Monodonta labio	PRJNA253054
Nemertopsis pamelaroeae	**PRJNA952238**
Nipponnemertes sp.	PRJNA254364
Ototyphlonemertes erneba	**PRJNA952238**
Paranemertes peregrina	PRJNA254365
Protopelagonemertes beebei	PRJNA254366
Riseriellus occultus	PRJNA254175
Tubulanus polymorphus	PRJNA263418
Tubulanus punctatus	PRJNA254065
*Capitella teleta**	Genome https://metazoa.ensembl.org/Capitella_teleta/Info/Index
*Lottia gigantea**	Genome http://metazoa.ensembl.org/Lottia_gigantea/Info/Index
*Monodonta labio**	PRJNA253054
*Myzostoma seymourcollegiorum**	PRJNA282832
